# Supplementary material for: Unveiling the Bioactive Potential of the Invasive Jellyfish Phyllorhiza punctata Through Integrative Transcriptomic and Proteomic Analyses
Source: Biomolecules. 2025 Aug 4;15(8):1121. doi: 10.3390/biom15081121 (PMC12383608; doi:10.3390/biom15081121)
Supplement: Supplementary file 1 [file biomolecules-15-01121-s001.zip › Suplementary Material/FileS3/PEPFOLD4/XP_065065086_11399/clusters.html]

 PEP-FOLD clusters


Download archive of all models  here   

|  |  |  |
| --- | --- | --- |
| # | file | sOPEP |
| # cluster 1 - Size: 88 |  |  |
|  | XP\_065065086\_11399-model1 | -9.23544 |
|  | XP\_065065086\_11399-model1.1 | -9.22199 |
|  | XP\_065065086\_11399-model1.2 | -9.22199 |
|  | XP\_065065086\_11399-model1.3 | -9.22199 |
|  | XP\_065065086\_11399-model1.4 | -9.22199 |
|  | XP\_065065086\_11399-model1.5 | -9.22199 |
|  | XP\_065065086\_11399-model1.6 | -9.22199 |
|  | XP\_065065086\_11399-model1.7 | -9.22199 |
|  | XP\_065065086\_11399-model1.8 | -9.22199 |
|  | XP\_065065086\_11399-model1.9 | -9.15795 |
|  | XP\_065065086\_11399-model1.10 | -9.15795 |
|  | XP\_065065086\_11399-model1.11 | -9.1537 |
|  | XP\_065065086\_11399-model1.12 | -9.1537 |
|  | XP\_065065086\_11399-model1.13 | -9.1537 |
|  | XP\_065065086\_11399-model1.14 | -9.14219 |
|  | XP\_065065086\_11399-model1.15 | -9.14219 |
|  | XP\_065065086\_11399-model1.16 | -9.14147 |
|  | XP\_065065086\_11399-model1.17 | -9.14147 |
|  | XP\_065065086\_11399-model1.18 | -9.14147 |
|  | XP\_065065086\_11399-model1.19 | -9.14147 |
|  | XP\_065065086\_11399-model1.20 | -9.14147 |
|  | XP\_065065086\_11399-model1.21 | -9.09355 |
|  | XP\_065065086\_11399-model1.22 | -9.09355 |
|  | XP\_065065086\_11399-model1.23 | -9.09355 |
|  | XP\_065065086\_11399-model1.24 | -9.07729 |
|  | XP\_065065086\_11399-model1.25 | -9.07729 |
|  | XP\_065065086\_11399-model1.26 | -9.07729 |
|  | XP\_065065086\_11399-model1.27 | -9.07729 |
|  | XP\_065065086\_11399-model1.28 | -9.07663 |
|  | XP\_065065086\_11399-model1.29 | -9.07458 |
|  | XP\_065065086\_11399-model1.30 | -9.06428 |
|  | XP\_065065086\_11399-model1.31 | -9.06428 |
|  | XP\_065065086\_11399-model1.32 | -9.06428 |
|  | XP\_065065086\_11399-model1.33 | -9.06428 |
|  | XP\_065065086\_11399-model1.34 | -9.05136 |
|  | XP\_065065086\_11399-model1.35 | -9.05136 |
|  | XP\_065065086\_11399-model1.36 | -9.05136 |
|  | XP\_065065086\_11399-model1.37 | -9.0211 |
|  | XP\_065065086\_11399-model1.38 | -9.00986 |
|  | XP\_065065086\_11399-model1.39 | -9.00974 |
|  | XP\_065065086\_11399-model1.40 | -9.00974 |
|  | XP\_065065086\_11399-model1.41 | -9.00974 |
|  | XP\_065065086\_11399-model1.42 | -9.00952 |
|  | XP\_065065086\_11399-model1.43 | -9.00171 |
|  | XP\_065065086\_11399-model1.44 | -9.0002 |
|  | XP\_065065086\_11399-model1.45 | -8.98547 |
|  | XP\_065065086\_11399-model1.46 | -8.98547 |
|  | XP\_065065086\_11399-model1.47 | -8.98547 |
|  | XP\_065065086\_11399-model1.48 | -8.98127 |
|  | XP\_065065086\_11399-model1.49 | -8.98127 |
|  | XP\_065065086\_11399-model1.50 | -8.96872 |
|  | XP\_065065086\_11399-model1.51 | -8.96872 |
|  | XP\_065065086\_11399-model1.52 | -8.95704 |
|  | XP\_065065086\_11399-model1.53 | -8.91915 |
|  | XP\_065065086\_11399-model1.54 | -8.91915 |
|  | XP\_065065086\_11399-model1.55 | -8.91915 |
|  | XP\_065065086\_11399-model1.56 | -8.91639 |
|  | XP\_065065086\_11399-model1.57 | -8.90885 |
|  | XP\_065065086\_11399-model1.58 | -8.90845 |
|  | XP\_065065086\_11399-model1.59 | -8.89187 |
|  | XP\_065065086\_11399-model1.60 | -8.89187 |
|  | XP\_065065086\_11399-model1.61 | -8.89187 |
|  | XP\_065065086\_11399-model1.62 | -8.87953 |
|  | XP\_065065086\_11399-model1.63 | -8.87809 |
|  | XP\_065065086\_11399-model1.64 | -8.86694 |
|  | XP\_065065086\_11399-model1.65 | -8.84833 |
|  | XP\_065065086\_11399-model1.66 | -8.82076 |
|  | XP\_065065086\_11399-model1.67 | -8.76829 |
|  | XP\_065065086\_11399-model1.68 | -8.76124 |
|  | XP\_065065086\_11399-model1.69 | -8.72385 |
|  | XP\_065065086\_11399-model1.70 | -8.70824 |
|  | XP\_065065086\_11399-model1.71 | -8.68947 |
|  | XP\_065065086\_11399-model1.72 | -8.67515 |
|  | XP\_065065086\_11399-model1.73 | -8.64722 |
|  | XP\_065065086\_11399-model1.74 | -8.6354 |
|  | XP\_065065086\_11399-model1.75 | -8.62877 |
|  | XP\_065065086\_11399-model1.76 | -8.60067 |
|  | XP\_065065086\_11399-model1.77 | -8.57543 |
|  | XP\_065065086\_11399-model1.78 | -8.56055 |
|  | XP\_065065086\_11399-model1.79 | -8.56034 |
|  | XP\_065065086\_11399-model1.80 | -8.56034 |
|  | XP\_065065086\_11399-model1.81 | -8.53007 |
|  | XP\_065065086\_11399-model1.82 | -8.50621 |
|  | XP\_065065086\_11399-model1.83 | -8.50254 |
|  | XP\_065065086\_11399-model1.84 | -8.50254 |
|  | XP\_065065086\_11399-model1.85 | -8.49911 |
|  | XP\_065065086\_11399-model1.86 | -8.36749 |
|  | XP\_065065086\_11399-model1.87 | -7.33885 |
| # cluster 2 - Size: 3 |  |  |
|  | XP\_065065086\_11399-model2 | -8.40554 |
|  | XP\_065065086\_11399-model2.1 | -8.08893 |
|  | XP\_065065086\_11399-model2.2 | -8.05174 |
| # cluster 3 - Size: 8 |  |  |
|  | XP\_065065086\_11399-model3 | -8.32123 |
|  | XP\_065065086\_11399-model3.1 | -8.32123 |
|  | XP\_065065086\_11399-model3.2 | -8.18348 |
|  | XP\_065065086\_11399-model3.3 | -7.95275 |
|  | XP\_065065086\_11399-model3.4 | -7.94764 |
|  | XP\_065065086\_11399-model3.5 | -7.85678 |
|  | XP\_065065086\_11399-model3.6 | -7.82537 |
|  | XP\_065065086\_11399-model3.7 | -7.6268 |
| # cluster 4 - Size: 1 |  |  |
|  | XP\_065065086\_11399-model4 | -6.92554 |
|  |  |  |
